# EGFL7 drives the evolution of resistance to EGFR inhibitors in lung cancer by activating NOTCH signaling

**DOI:** 10.1038/s41419-022-05354-y

**Published:** 2022-10-29

**Authors:** Yubo Wang, Pu Chen, Man Zhao, Hongxin Cao, Yuelei Zhao, Meiju Ji, Peng Hou, Mingwei Chen

**Affiliations:** 1 Department of Respiratory Medicine, The First Affiliated Hospital of Xi’an Jiaotong University, Xi’an, 710061 Shaanxi PR China; 2 Key Laboratory for Tumor Precision Medicine of Shaanxi Province, The First Affiliated Hospital of Xi’an Jiaotong University, Xi’an, 710061 PR China; 3 Department of Endocrinology, The First Affiliated Hospital of Xi’an Jiaotong University, Xi’an, 710061 Shaanxi PR China; 4 Center for Translational Medicine, The First Affiliated Hospital of Xi’an Jiaotong University, Xi’an, 710061 Shaanxi PR China

**Keywords:** Non-small-cell lung cancer, Cancer therapeutic resistance, Mechanisms of disease

## Abstract

Accumulating evidence supports evolutionary trait of drug resistance. Like resilience in other systems, most tumor cells experience drug-tolerant state before full resistance acquired. However, the underlying mechanism is still poorly understood. Here, we identify that EGF like domain multiple 7 (*EGFL7*) is a responsive gene to epidermal growth factor receptor (EGFR) kinase inhibition during a period when tumors are decimated. Moreover, our data reveal that the adaptive increase of EGFL7 during this process is controlled by the depression of nonsense-mediated mRNA decay (NMD) pathway. Upregulation of EGFL7 activates NOTCH signaling in lung cancer cells, which slows down the decrease of c-Myc caused by EGFR inhibition, thereby helping the survival of cancer cells. Our data, taken together, demonstrate that EGFL7 is a driver gene for resistance to EGFR kinase inhibition, and suggest that targeting EGFL7/NOTCH signaling may improve the clinical benefits of EGFR inhibitors in patients with *EGFR* mutant tumors.

## Introduction

Although the use of epidermal growth factor receptor (EGFR) inhibitors in *EGFR*-mutant non-small-cell lung cancer (NSCLC) has led to a major clinical breakthrough, eventual resistance to these inhibitors limits their further clinical benefits as happened in conventional chemotherapy [1]. Generally, drug resistance can be roughly categorized into primary resistance and acquired resistance. Primary resistance refers to a situation where tumors do not have drug target or have preexisting certain mutations, such as *EGFR* T790M mutation [2]. Acquired resistance describes a situation where tumors initially respond to drugs such as EGFR inhibitors, then relapse and progress after a period of exposure [3]. The activation of alternative pathway is a typical example of acquired resistance [4].

Although onset of new mutants or histology demonstrated in clinical samples [5] seems to support that primary resistance plays a major role, those data can also be explained by either parallel evolution [6] or adaptive mutability caused by inhibition of EGFR [7]. Besides, clinical data often lack serial biopsies and transcriptome data, thereby dampening their ability to determine the dynamics of resistance development [8] and elucidate the underlying mechanism [9]. Thus, acquired resistance may be at least of equal importance to primary resistance. Acquired resistance defines a dynamic process from sensitive status to resistance and renders a new therapy target in addition to multifactorial resistance [10]. Upon treatment, the fate of sensitive cells may be a stochastic process. That is, most cells will die while a constant proportion of cells gain a dormant, non-dividing state and survive the treatment [8]. Both extracellular matrix (ECM) [11] and epigenetic change [8] may contribute to this state. The remaining cells, namely persister cells, then will follow different paths to irreversible resistance [12]. Alternatively, persister cells can also regain drug sensitivity upon treatment withdraw. Indeed, some retrospective clinical data confirm re-challenge is effective [13], especially in patients without known mechanisms of resistance [14]. Thus, targeting seemingly common path before irreversible resistance may convey more clinical benefits, especially considering diverse resistant mechanisms confirmed [15]. Although persister cells express some stem cell markers and have special redox levels compared with parental cells [16], the pathway underlies the formation of persister cells from inhibition of EGFR is still poorly understood.

Based on an assumption that “driver genes” of acquired resistance should respond robustly to the inhibition of EGFR and persist during the evolution to resistance, the present study demonstrates that EGFL7 is upregulated under the perturbation of EGFR kinase and its responsive increase buffers the inhibition of EGFR, thereby promoting the transition of parental cells to persistent cells and eventually resistant cells. Coined terms from tumorigenesis, these results indicate that EGFL7 is a driver gene of acquired resistance to EGFR inhibitors.

## Materials and methods

### Cell lines

Human NSCLC cell lines PC9, HCC827 and derived osimertinib-resistant PC9OR cell lines were gained from Prof. Shiyong Sun (Emory University School of Medicine and Winship Cancer Institute, Atlanta, GA). These cells were routinely cultured in RPMI 1640 containing 10% fetal bovine serum (FBS) at 37 °C with 5% CO_2_.

### Animals

The animal experiments in this study were approved by the Laboratory Animal Administration Committee of Xi’an Jiaotong University and performed according to the Guidelines for Animal Experimentation of Xi’an Jiaotong University. Male athymic (nu/nu) mice, aging between 6 to 8 weeks, were purchased from SLAC laboratory Animal Co., Ltd. (Shanghai, PR. China), and housed under specific pathogen-free conditions. The simple randomization was used to categorize mice. The indicated cells at 1 × 10^6^ in serum-free medium were injected subcutaneously into the flank region of nude mice to establish the tumor xenografts model. Next, these mice were administrated with 3 mg/kg/day osimertinib (gavage) alone or in combination with 1 mg/kg/day DBZ (intraperitoneally) when the neoplasms reached sizes of about 50–100 mm^3^ (calculated by 0.5*length*width*width). Tumor volume was calculated by the above formula. The mice were then sacrificed after 26 days and the tumor tissues were stripped and weighed for further experiments. No statistical methods were used to determine sample size and the investigators were blind to group allocation during measurement.

### Immunohistochemistry

Xenografts fixation and immunohistochemistry were performed as described previously [17, 18]. Briefly, 4 µm paraffin sections were dewaxed with xylene, rehydrated with graded alcohol series and heated with citrate buffer. After that, Ki-67 (1: 100 dilutions, BD Pharmingen™), biotin conjugated secondary antibody and streptavidin-HRP were applied, and DAB was then added to show positive cells.

### Knockdown and ectopic expression of EGFL7

Control siRNA (si-NC) and specific siRNAs targeting EGFL7 (si-EGFL7-1 and si-EGFL7-2) were purchased from Guangzhou RiboBio Co., Ltd. (Guangzhou, China). Lentivirus expressing sh-RNA targeting EGFL7 (sh- EGFL7) and control lentivirus (sh-NC) were purchased from HanBio Co., LTD (Shanghai, China). About 5 × 10^4^ cells per well were seeded in 12-well plate and cultured in CO_2_ incubator (Thermo Fisher) overnight. Next, cells were transfected with si-EGFL7-1/2 or si-NC using X-tremeGENE™ siRNA Transfection Reagent (Roche) or infected with the above lentiviruses according to the manufactory’s guide. The sequences of the siRNAs and shRNAs were presented in Supplementary Table S1.

The full-length open reading frame (ORF) of human EGFL7 with MYC tag was amplified and cloned into plasmid pcDNA3.1(-) with using the primers bellow. Next, 1 µg EGFL7 plasmid or empty plasmid and X-tremeGENE HP DNA Transfection Reagent (Roche) were added to 12-well plate (1 × 10^5^ cells/well) as the manufactory’s guide. The sequences used for plasmid construction were 5’-GCT GGA TAT CTG CAG AAT TCG CCA CCA TGA GGG GCT CTC AGG-3’ (forward) and 5’-TGC AAG AAA GAC TCG GAG CAG AAA CTC ATC TCA GAA GAG GAT CTG TGA GGA TCC GAG CTC GGT ACC AAG C-3’ (reverse).

### Cytotoxicity assay

About 2000–4000 cells per well were cultured in 96-well plates and measure by SRB or MTT assay as described previously [19, 20].

### Generation of persister cells

PC9 cells were cultured in 35 mm dishes and treated with 1 µM osimertinib for 9 days. Fresh medium containing drug was then replaced at about 48, 84, 120, 168 and 216 h, respectively. Next, the persister cells were washed with phosphate buffered saline(PBS) twice, fixed for 15 min with methanol and then stained with 1 g/ml Crystal Violet. After washing and air dry, cell number was counted under light microscope.

### Cell cycle analysis

About 2 × 10^5^ PC9OR cells per well were seeded into 6-well plate and cultured overnight. Next morning, 2 µM osimertinib or vehicle was added and further cultured for 48–72 h. Cells were then detached with trypsin-EDTA (Byotime), stained with propidium iodide (Sigma-Aldrich) and analyzed by BD FACSCalibur™ flow cytometer (BD Biosciences).

### Competitive fitness analysis

About 1 × 10^5^ PC9 cells and green fluorescent protein (GFP)-labeled PC9 cells were dropped in 60 mm dishes with or without mixture and cultured in CO_2_ incubator. Next morning, cells were checked under light microscope to make sure the formation of evenly distribution or two distinct clumps. Following this, cells were treated with 1 µM osimertinib or vehicle for 36–48 h, and then analyzed by BD FACSCalibur™ flow cytometer (BD Biosciences).

### RNA sequencing

Total RNA was extracted using TRIzol™ (Invitrogen^TM^) and then qualified using Agilent 2100 bioanalyzer (Agilent). Next, 0.2 µg RNA and TruSeq® Stranded kit (Illumina) was used to prepare RNA libraries according to manufactory’s guide. Libraries were then sequenced using Illumina HiSeq at 100 bp, and the clean reads were obtained by filtering out rRNA, adapter and low-quality reads using SOAP (ver. 1.5.2) [21]. Data were restored at GEO under accession number GSE201549.

### Transcriptome analysis

Patients with lung adenocarcinoma receiving the treatment of EGFR inhibitors were categorized into two groups: response group (Complete Response) and no response group (Clinical Progressive Disease), and raw count data were then extract from TCGA database. We obtained raw sequence data through Galaxy (SRP066956) whose raw counts data cannot directly downloaded from GSE75602 [22] and sequence data were then mapped to Genome Reference Consortium Human Build 37 (GRCh37) using STAR package [23]. The raw count data were obtained using HTseq package [24]. Differential expressed genes of NR. group vs. R. group, PC9 vs. PC9OR cells and persister vs. naive cells (GSE75602) were calculated using DEGseq (ver. 1.48.0) package [25] in R.

To identify *EGFL7* as a late response gene to EGFR kinase inhibition. As for data from GSE43288, GSE178755, GSE114647, GSE65420, raw data were obtained from GEO database. Raw counts data from GSE75602 were obtained as described. These raw data were then normalized using limma (ver. 3.46.0) package [26] according to user’s guide. As for microarray data, normalized data of GSE45891 were obtained from GEO database using GEOquery (ver. 2.58.0) package [27], while raw data of GSE115864, GSE20854, GSE512512, GSE68954 and GSE106151 were obtained from GEO database and affy (ver. 1.68.0) package [28] was used to get normalized expression matrix. The limma (ver. 3.46.0) package [26] was then used to calculate relative expression change of *EGFL7*.

### Gene set enrichment analysis

We obtained raw data from GEO database (GSE159095, GSE168642, GSE85986, GSE126933, GSE134401, GSE61999, GSE62000, GSE71769, GSE38054, GSE61869, GSE61870 and GSE61871). We obtained raw sequence data from Galaxy (GSE87615) [22] and corresponding raw counts data were obtained as described above. These raw data were normalized using limma (ver. 3.46.0) package [26] according to user’s guide. As for microarray data, raw data obtained from GEO database (GSE134114, GSE104260, GSE74631, GSE6495, GSE7067, GSE27424, GSE34602 and GSE54378) and affy (ver. 1.68.0) package [28] was used to get normalized expression matrix according to user’s guide. Also, we obtained normalized data from GEO database (GSE30288, GSE45750 and GSE53203) using GEOquery (ver.2.58.0) package [27]. Besides, 14 c-Myc-related gene sets were obtained from the Molecular Signatures Database (MSigDB) [29] (Supplementary Table S2). Normalized enrichment scores were then calculated using GSEA software (ver. 4.2.0) [30] with default settings.

### Single cell sequence analysis

Raw counts data were obtained from GSE150949. Expression of *EGFL7* and *p* values between groups were then calculated using MAST (ver. 1.16) package according to user’s guide. NOTCH signature was also obtained from supplements [31]. Besides, mean NOTCH signaling score per cell was calculated as described previously [32], and *p* values of NOTCH signaling score were then calculated using Student’s *t* test.

### Specific transcript PCR

Total RNA was extracted using TRIzol™ (Invitrogen^TM^) and reverse-transcribed into cDNA using PrimeScript™ RT Reagent Kit with gDNA Eraser (TaKaRa). Specific *EGFL7* transcripts were amplified using Platinum™ II Taq Hot-Start DNA Polymerase (Invitrogen™) and specific primers (forward: 5’-CTA GGG TCC ATC TCC AGT CC-3’; reverse: 5’-CCA ACA CCA GAA GCC ACA TCA G-3’) according to manufactory’s guide. Secondary PCR was done with resulting production. The presence of specific *EGFL7* transcripts was presented by DNA Electrophoresis.

### Western blot analysis

Whole-cell lysates were prepared in RIPA lysis buffer containing PMSF and protease inhibitor cocktail, 30–60 µg proteins were then subjected to western blot analysis loaded into SDS-PAGE gels and transferred to polyvinylidene difluoride membrane(Roche) as described previously [33]. The antibody information was presented in Supplementary Table S3.

### Statistical analysis

Each in vitro experiment was repeated three times and in vivo experiment was done once with at least three biological replicate. No statistical methods were used to determine sample size. The results are shown as mean ± standard error of the means (SEM) other than specified. The specific statistical tests used to determine differences are selected according to data traits and described in figure legends. Briefly, we use two-tailed Welch Two Sample *t*-test for comparing difference of mean of two independent sample, one-way ANOVA with Tukey multiple comparisons for 3 or more independent samples analysis, two-way ANOVA for curve analysis, linear models and MA-plot-based methods for transcriptome analysis and Zero-inflated regression for single-cell transcriptome analysis. *p* < 0.05 was considered as significant and *p* values were calculated using R.

## Results

### EGFL7 functions in cancer resistance to EGFR inhibitors

**Fig. 1:**
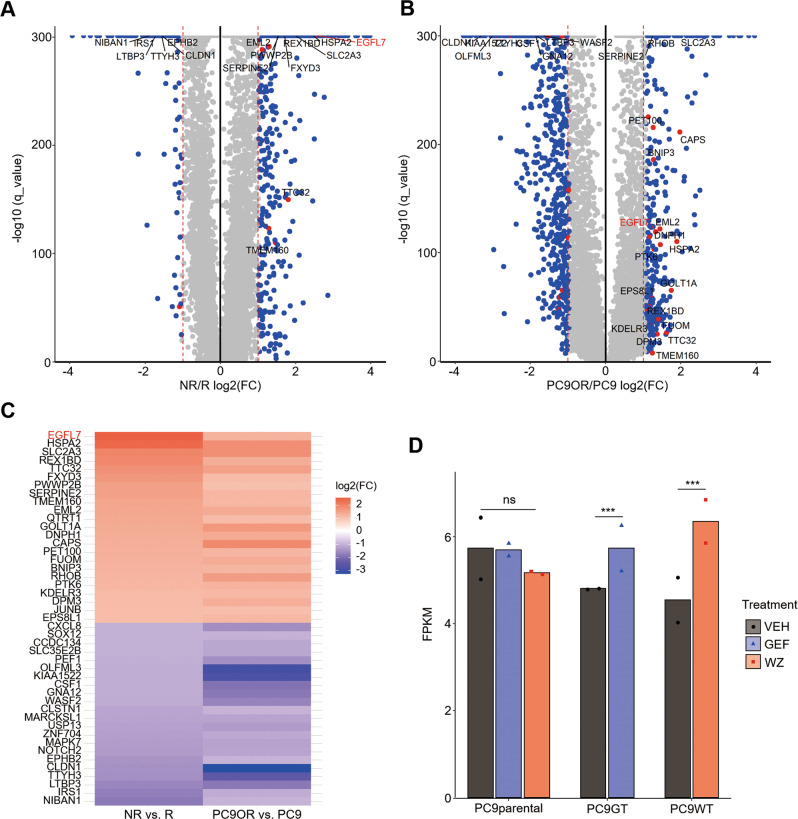
EGFL7 correlates to EGFR inhibitors resistance in cancer. **A** Volcano plot representing differential expressed genes between non-responders (NR) and responders (R) to EGFR inhibitors (data from TCGA database). **B** Volcano plot representing differential expressed genes between PC9OR cells and PC9 cells. Common genes in **A** and **B** are represented as red dots. **C** Heat map showing relative abundance of common differential expressed genes in **A** and **B**. **D** Expression of *EGFL7* transcripts in PC9 cells (PC9parental), gefitinib-derived PC9 tolerant cells and WZ4002-derived PC9 tolerant cells upon vehicle, EGFR inhibitor gefitinib (GEF) or EGFR inhibitor WZ4002 (WZ) treatment (GSE75602). Data are shown as mean values of two replicates. *p* values are calculated by DEG package in R (see method). (ns, *p* > 0.05; ***p < 0.001).

To clarify the dynamics of drug resistance, we first selected patients who received the treatment of EGFR inhibitors from The Cancer Genome Altas database (TCGA), and divided them into response (complete response) and nonresponse (clinical progressive disease) groups (Supplementary Table S4). Next, we analyzed the transcriptome of these two groups and got 632 highly expressed genes in no responders (Fig. 1A) based on the threshold with >2-fold change and *q* value <0.001. Meanwhile, we obtained 369 genes upregulated in osimertinib-resistant PC9OR cells (Fig. 1B) with the same threshold by comparing the gene expression profiles of PC9 cells and derived PC9OR cells. After that, we overlapped these tow differential expressed gene sets and found 23 upregulated genes, among which *EGFL7* ranked first (Fig. 1C). In addition, we found a dataset showing that *EGFL7* was significantly elevated upon EGFR inhibition in PC9 tolerant cells rather than PC9 parental cells compared with vehicles (Fig. 1D) [12].

**Fig. 2:**
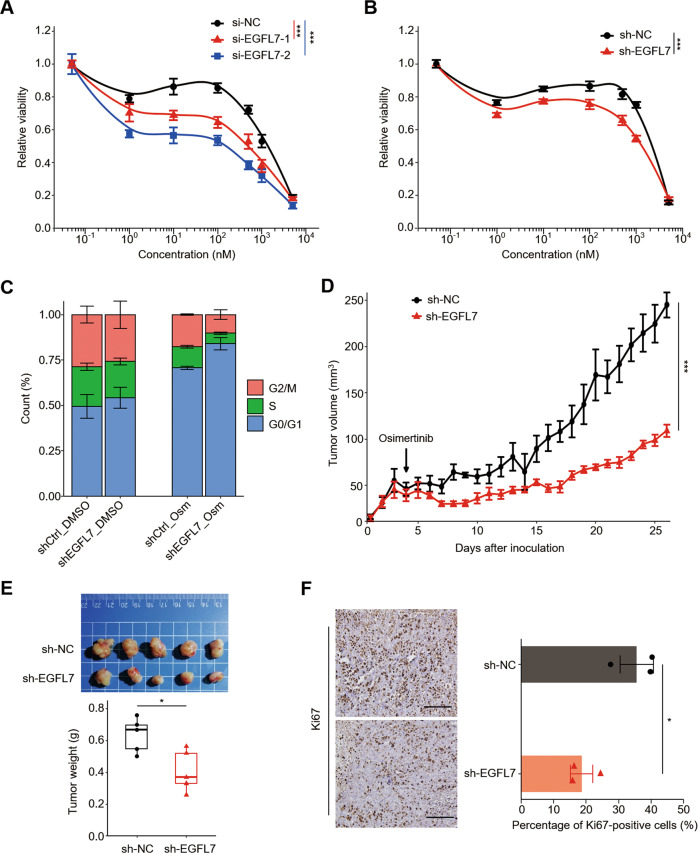
EGFL7 promotes resistance to EGFR inhibitors in acquired resistance phase. Survival curve of osimertinib-resistant PC9 (PC9OR) cells with the indicated si-RNAs (**A**) or sh-RNAs (**B**) in different concentration of osimertinib treatment for 72 h. Data present as average of four identically wells of percentage of surviving cells to 6 wells of vehicle-treated control. **C** Cell cycle distribution of PC9OR cells with the indicated sh-RNAs treated with 2 µM osimertinib or vehicle. Data present as mean ± standard deviation (SD) of three biological replicates. **D** Growth curves of tumor xenografts derived from EGFL7-knockdown PC9OR cells or control cells treated with 3 mg/kg Osimertinib. **E** Images of the indicated xenograft tumors (upper panel) and statistical analysis of tumor weight (lower panel). **F** Left panel showing the representative images of Ki67 staining in the indicated xenograft tumors, and right panel showing statistical analysis of the percentage of Ki67 positive cells. Scale bar: 200 µm. The data in right panel (**F**) are average value of three biological replicates. The data in **E** are average value of five biological replicates. The median line in **E** represents median values. The top and bottom of box in **E** are 25th and 75th percentiles. Data in **A**, **B**, **D**–**F** are shown as mean ± standard error of the mean (SEM). *p* values in **A**, **B**, **D** are calculated by two way ANNOVA. *p* values in **E** and **F** are calculated by unpaired two-tailed Student’s *t t*est (*p < 0.05; ***p < 0.001).

To valid the role of EGFL7 in resistance to EGFR inhibitors, we first knocked down its expression in PC9OR cells by two specific siRNAs (si-EGFL7-1 and si-EGFL7-2), and found that EGFL7 knockdown increased cellular response to EGFR inhibitor osimertinib compared with the control (Fig. 2A), as measured by 15% and 25% decrease in area under curve (AUC). To exclude possible off-target effect [34], we further confirmed this effect (Fig. 2B) using shRNA specifically targeting EGFL7. Moreover, EGFL7 knockdown caused a higher proportion of PC9OR cells arrested in G_0_/G_1_ phase upon osimertinib treatment relative to the control, while did not change cell cycle distribution in untreated cells (Fig. 2C). Correspondingly, ectopic expression of EGFL7 led 4% increase in AUC (Supplementary Fig. S1A) and a lower proportion of G_0_/G_1_ cell cycle-arrested PC9OR cells (Supplementary Fig. S1B). Next, we established the xenograft tumor model by subcutaneously injecting EGFL7-knockdown PC9OR cells and control cells into the armpit of nude mice, and found that knockdown of EGFL7 resulted in a slower growth rate of xenograft tumors when mice were treated with osimertinib, as measured by tumor volume (Fig. 2D), tumor weight (Fig. 2E) and the levels of proliferative marker Ki67 (Fig. 2F). These results, taken together, indicate that EGFL7 may function in resistance to EGFR inhibitors.

### *EGFL7* is a late response gene to EGFR kinase inhibition

**Fig. 3:**
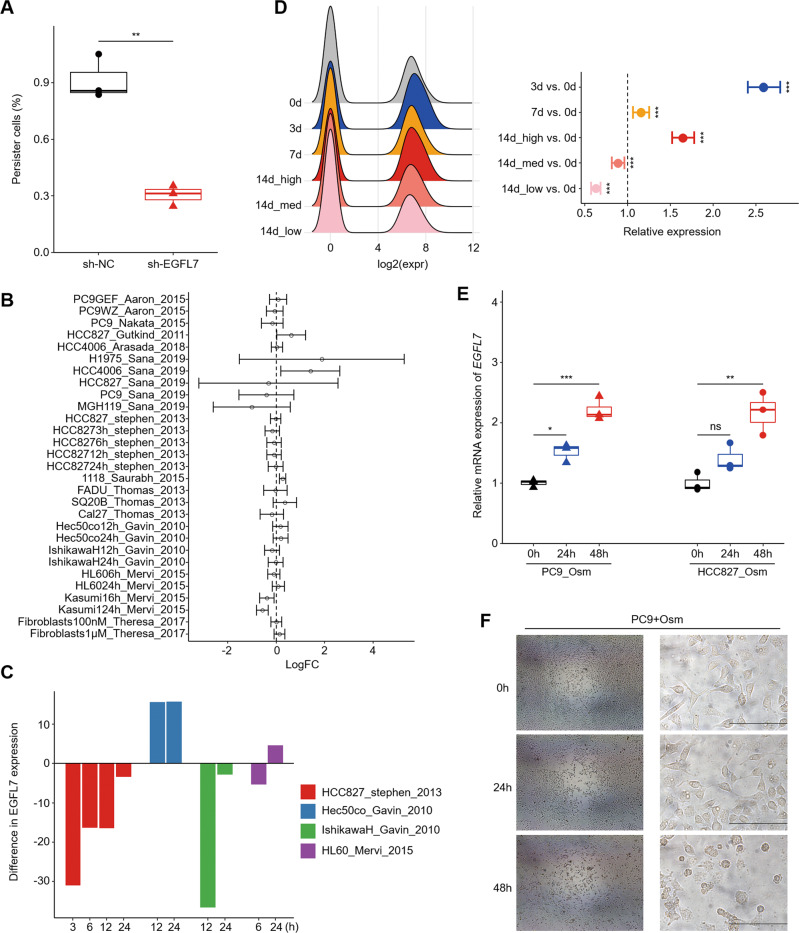
Validation of EGFL7 as a late response gene to EGFR kinase inhibition. **A** The proportion of PC9 cells remained after a 9-day osimertinib treatment to seeding number. The median line represents median values. The top and bottom of box are 25th and 75th percentiles. **B** Relative expression of *EGFL7* in cancer cells treated with EGFR inhibitors to the control in indicated studies. Dashed line presents no change (fold change equal to 1). Point and error bars present mean value and 95% confidence interval (see methods). **C** Difference in *EGFL7* expression upon different treatment duration of EGFR inhibitors in the indicated datasets from **B**. **D** Relative *EGFL7* expression in groups of PC9 single cells (GSE150949, see methods). Dashed line presenting no change (fold change equal to 1) in right panel. **E** Relative *EGFL7* expression in PC9 and HCC827 cells treated with osimertinib for the indicated time duration. Point and error bars present mean value and 95% confidence interval in **D** and mean ± standard error of the mean (SEM) in **E**. *p* values are calculated by MAST package (**D**). **F** Representative light microscopic images of PC9 upon different osimertinib treatment duration. Scale bar: 200 µm. *p* values are calculated by two-tailed Student’s *t* test (ns, *p* > 0.05; *p < 0.05; **p < 0.01; ***p < 0.001) otherwise specified.

Based on the hypothesis that acquired resistance is a result of dynamic processes from naive status, we next explored the role of EGFL7 in early phase. As shown in Supplementary Fig. S1C, knocking down EGFL7 in PC9 cells decreased AUC by 2% when exposed to different concentration of osimertinib compared with the control. More importantly, the proportion of resident cells under high concentration of osimertinib was lower in EGFL7-knockdown cells compared with control cells. Conversely, ectopic expression of EGFL7 slightly increased the proportion of PC9 resident cells compared with the control although their difference was not statistically significant (Supplementary Fig. S1D). To further determine the role of EGFL7 in the transition of parental cells to persistent cells, we treated EGFL7-knockdown PC9 cells and control cells with 1 μM osimertinib for 9 days, and found that EGFL7 knockdown significantly reduced the proportion of persister cells compared with the control (Fig. 3A).

It is the key to understand cell signaling and genetic regulatory pathways involved in human diseases or clinical manifestations by determining how cells respond to perturbing agents. We thus searched Gene Expression Omnibus (GEO) repository to support EGFL7 as a responsive gene to EGFR inhibitors. Although the datasets showed inconsistent results (Fig. 3B), we noticed that the expression of *EGFL7* increased with treatment duration in most situations (Fig. 3C). This was supported by the data showing that *EGFL7* was significantly upregulated in drug-tolerant cells when exposed to EGFR inhibitors (Fig. 1D). Similarly, a recent study profiled the expression of PC9 persister cells by single-cell RNA sequencing, at four time points (days 0, 3, 7 and 14) during osimertinib treatment. While, on day 14, persister cells were divided into three subsets: cycling (14d_low), moderately cycling (14d_med) and non-cycling (14d_high) persisters [31]. The results showed that *EGFL7* expression was significantly elevated upon osimertinib treatment at days 3, 7 and 14 (only main subset) compared with day 0 (Fig. 3D). Intriguingly, the rare subsets (14d_low and 14d_med), regarded as subsets somehow insensitive to drug treatment, failed to show an increased expression of *EGFL7*. Based on these findings, we speculate that *EGFL7* may be a late response gene to EGFR kinase inhibition. As supported, our data showed that *EGFL7* expression was higher in PC9 and HCC827 cells treated with osimertinib for 48 h than those with a 24-h treatment (Fig. 3E). Notably, PC9 cells seemed intact until treatment duration was up to about 36–48 h (Fig. 3F), indicating genes that were regulated in this period were more probable to help the survive of cancer cells upon drug treatment. These results, taken together, support the above conclusion.

### Depression of nonsense-mediated mRNA decay (NMD) causes the late response of EGFL7 to EGFR inhibitors

Considering turnover rates of mRNAs [35] and proteins [36], we speculate that the late response of EGFL7 should be indirectly regulated by EGFR kinase inhibition. To determine the underlying indirect mechanism, we first analyzed transcription factors whose expression was not altered in PC9 parental cells treated with osimertinib for 24 h, but differentially expressed in PC9 persister cells treated with osimertinib for 24 h, and found 37 candidate genes (Supplementary Fig. S2). We then filtered these genes by motif prediction provided in Eukaryotic Promoter Database [37] and conservative IDR peaks in *EGFL7* promoter from Encyclopedia of DNA Elements [38] or other published chromatin immunoprecipitation sequencing datasets, and eventually identified *GLIS2* as a target gene. However, further searching in Gene References Into Functions implied that GLIS2 generally functioned as repressors, thus it is unlikely to upregulate EGFL7.

**Fig. 4:**
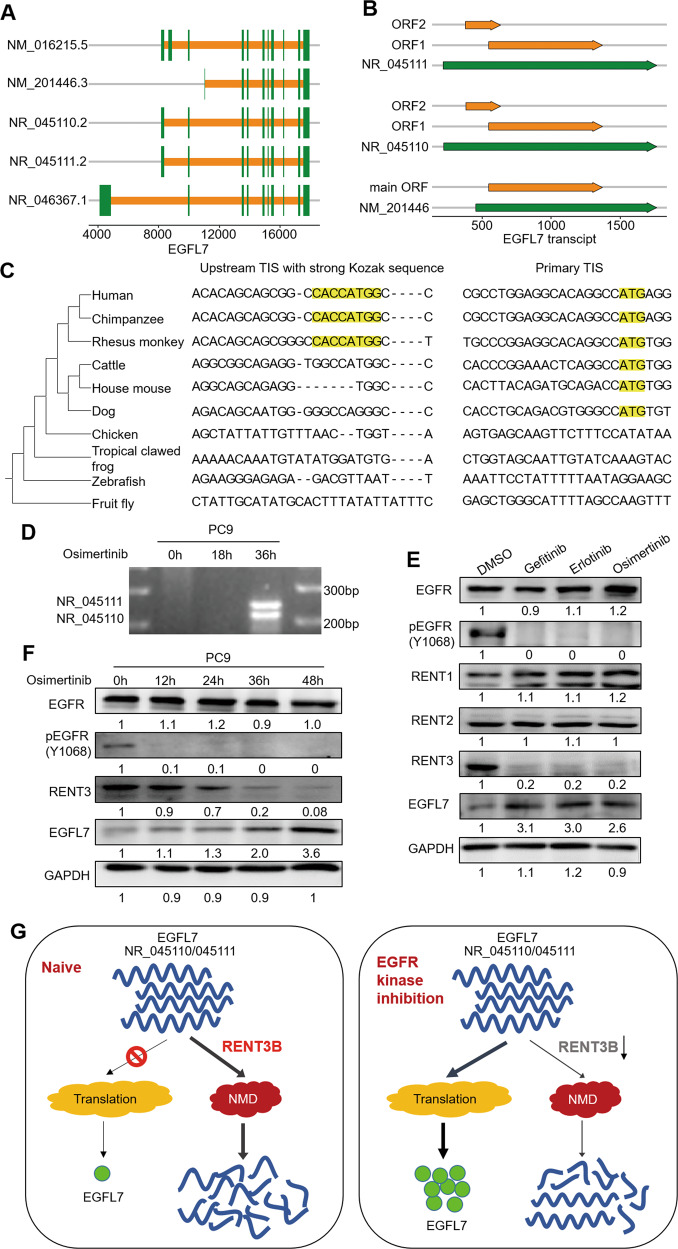
Long-term inhibition of EGFR kinase leads to decreased decay of *EGFL7* transcripts. **A**, **B** Schematic of *EGFL7* transcripts and open reading frames (ORF) in certain transcripts. **C** Two small alignment regions corresponding to two translation initiation sites (TISs) of *EGFL7* in ten species. The evolutionary tree obtained from NCBI Taxonomy database is presented on the left. **D** The electrophoresis presenting specific *EGFL7* transcript in PC9 cells treated with osimertinib for the indicated time points. **E** Western blot analysis showing the levels of EGFR, pEGFR, RNET1, RNET2, RNET3 and EGFL7 in response to the indicated EGFR kinase inhibitors. **F** Western blot analysis showing the level of EGFR, pEGFR, RNET3 and EGFL7 in PC9 cells treated with osimertinib for the indicated time points. GAPDH was used as a loading control. **G** Schematic of declined decay of *EGFL7* transcripts in response to EGFR kinase inhibition.

We next attempted to identify the factors that affect mRNA decay. Notably, EGFL7 has five transcripts in well-annotated NCBI RefSeq database (Fig. 4A), among which three transcripts are annotated as non-coding due to the presence of an upstream ORF that renders those transcripts candidates for NMD pathway, which degrades select mRNAs with translation termination codons positioned in suboptimal contexts [39]. Intriguingly, we detected the same ORF encoding EGFL7 protein in these transcripts using ORFfinder provided by NCBI with default settings (Fig. 4B), indicating that they have the potential to be translated if not decayed via NMD pathway. We speculate that these rapid decayed non-coding transcripts serves a reservoir of EGFL7. To prove this, we first obtained orthologous EGFL7 sequences from NCBI HomoloGene database and aligned these 10 genes using MEGA [40]. The results showed that upstream translation initiation site (TIS) with strong Kozak sequence was conserved among Catarrhini parvorder, while primary TIS was conserved among Boreoeutheria clade (Fig. 4C), suggesting the physiological function of seemingly wasted events.

We attempted to distinguish EGFL7 main transcript (NM_201446) and other transcripts (NM_016215, NR_45110 and NR_45111) by designing specific primers. The results showed that NR_45110 and NR_45111 exited only in PC9 cells with a 36-h osimertinib treatment (Fig. 4D). We then analyzed the expression of three core proteins (RENT1, RENT2 and RENT3) of NMD pathway in PC9 cells, and found only RENT3 was obviously downregulated when exposed to osimertinib compared with the control (Fig. 4E). Also, we measured the expression of RENT3 at five time points (0, 12, 24, 36 and 48 h) during osimertinib treatment, and found that the expression pattern of RENT3 was highly consistent with the dynamics of *EGFL7* transcripts and proteins (Fig. 4F). Besides, a previous study has implied that alternative TIS selection is a widespread phenomenon and that TIS used by one transcript can change in response to some stimulus [41]. Collectively, our data indicate that the late response of EGFL7 to EGFR kinase inhibition is at least partly caused by decreased decay of some isoforms of EGFL7 (Fig. 4G).

### EGFL7 buffers EGFR kinase inhibition by activating NOTCH signaling

**Fig. 5:**
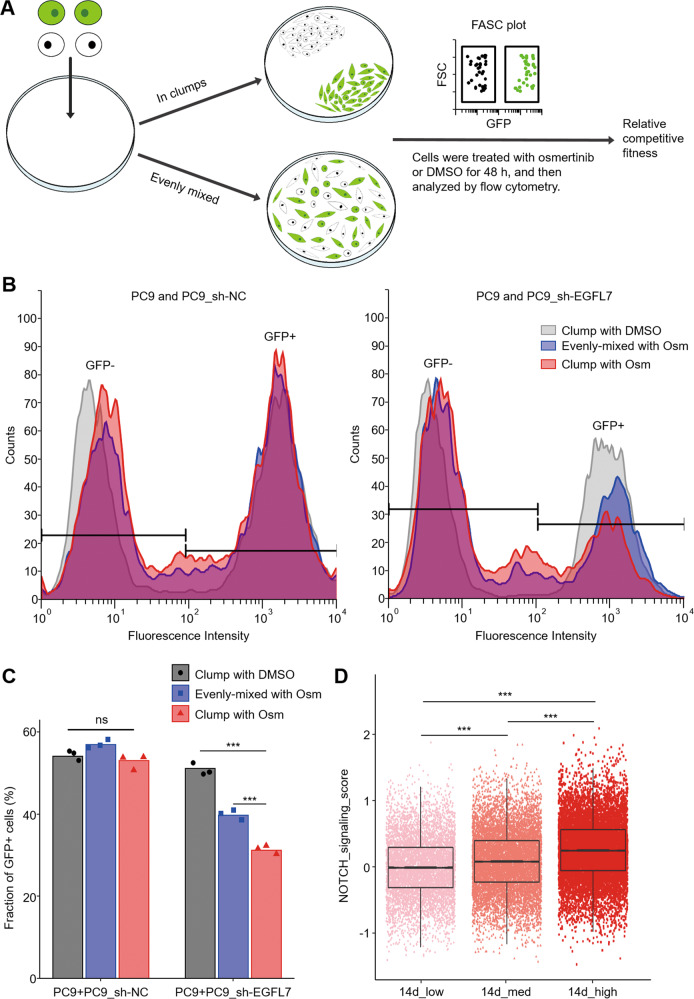
EGFL7 activates NOTCH signaling extracellularly. **A** Schematic of experimental workflow for cell competition assay to access the relative fitness in osimertinib (Osm) exposure. **B** FACS plot presenting cell number across different fluorescence intensity in the mixture of PC9 cells and EGFL7-knockdown PC9 cells (right panel) or control cells (PC9_ sh-NC, left panel). **C** Bar plot presenting the statistic results from **B**. **D** Boxplot presenting NOTCH signaling scores in low, medium and high mCherry expression PC9 subgroups with a 14-day osimertinib treatment (GSE150949, *N* = 26,990, see methods). FACS indicates fluorescent-activated cell sorting. *p* values are calculated by one-way ANNOVA with Tukey multiple comparisons (ns, *p* > 0.05; ***p < 0.001).

We sought to determine the mechanism underlying the buffer effect of EGFL7 on EGFR inhibitors. Considering that EGFL7 is recognized as a secretory protein but its vascular tubulogenesis function requires its vicinity of endothelial cells [42], we mixed naive PC9 cells and GFP-labeled EGFL7-knockdown PC9 cells (PC9_sh-EGFL7) or control cells (PC9_sh-NC) in either evenly distributed pattern or clumps and then exposed them to 1 μM osimertinib for 48 h (Fig. 5A). The results showed that the fraction of EGFL7-knockdown PC9 cells in clumps and evenly distributed pattern declined by about 40% and 22% as measured by flow cytometry when exposed to osimertinib compared with the control (DMSO), respectively (Fig. 5B, C). That is, GFP-labeled EGFL7-knockdown PC9 cells (PC9_sh-EGFL7) showed less relative fitness in clumps compared with those in evenly distributed pattern when exposed to osimertinib. On the contrary, the relative fitness of GFP-labeled control cells (PC9_sh-NC) was similar in above situations (Fig. 5B, C). These data, taken together, suggest that EGFL7 buffers EGFR kinase inhibition probably through interacting with extracellular proteins.

**Fig. 6:**
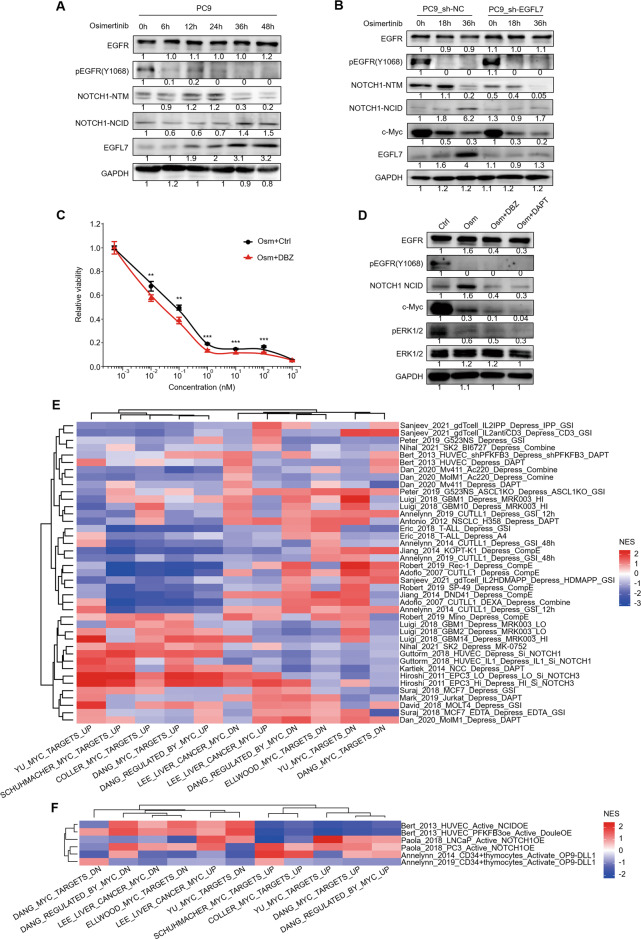
EGFL7-mediated activation of NOTCH signaling buffers EGFR kinase inhibition. **A** Western bolt analysis showing the levels of EGFR, pEGFR, the first cleaved product (NTM) and active form (NCID) of NOTCH1 as well as EGFL7 in PC9 cells treated with 1 µM osimertinib (Osm) for the indicated time points. **B** Western bolt analysis showing the levels of EGFR, pEGFR, NOTCH1-NTM, NOTCH1-NCID, c-Myc and EGFL7 in EGFL7-knockdown PC9 cells and control cells treated with 1 µM osimertinib (Osm) for the indicated time points. GAPDH was used as a loading control. **C** Survival curves of PC9 cells treated with a combination of different concentrations of osimertinib (Osm) and vehicle control (DMSO) or NOTCH signaling inhibitor dibenzazepine (DBZ) for 72 h. Data present as average of four identically wells of percentage of surviving cells to vehicle-treated control. **D** Western blot analysis showing the levels of change of EGFR, pEGFR, NOTCH1-NCID, c-Myc, pERK and ERK1/2 in PC9 cells treated with the indicated treatments. GAPDH was used as a loading control. **E**, **F** Heat maps presenting the normalized enrichment score of the indicated gene sets in the indicated experiments when the NOTCH signaling was activated or repressed (see methods). Data are shown as mean ± SEM. *p* values are calculated by unpaired two-tailed Student’s *t* test (**p < 0.01; ***p < 0.001).

We nest searched the interactome of EGFL7 in HuRI [43] and BioGRID [44] databases, and got 9 and 74 interactors (Supplementary Tables S5 and 6). Among them, considering their subcellular localizations, only notch receptor 1(NOTCH1) may induce the buffer effect of EGFL7 on EGFR kinase inhibition. In addition, a previous study revealed a positive correlation between the expression of *EGFL7* and the activation of NOTCH signaling pathway in subsets from a 14-day osimertinib treated PC9 persister cells when most cells formed clones with distinct ECM (Fig. 5D) [31]. Besides, another study has indicated that EGFL7 interacts with extracellular domain of NOTCH1 receptor [45]. Thus, we speculate that EGFL7 may activate NOTCH signaling pathway, thereby contributing to its buffer effect on EGFR kinase inhibition. To validate this, we first determined the temporal pattern of NOTCH signaling in PC9 cells exposed to osimertinib by western blot analysis. It is well known that the activation of Notch1 receptor requires two proteolytic cleavage events [46]. However, osimertinib treatment seemed to uncouple these two processes. As shown in Fig. 6A, the product of first proteolytic cleavage events, named transmembrane/intracellular region(NTM), increased in first 24 h, and then declined in response to osimertinib, whereas the final products, named the intracellular domain (NCID) that is the region that actives downstream signaling, began increasing at about 36 h after osimertinib treatment, which was consistent with the pattern of EGFL7. Thus, we hypothesized that early increase of NTM region was caused by other factors, while the late increase of EGFL7 led to the late increase of NCID and NOTCH signaling activation. To support this, we compared NOTCH signaling between EGFL7-knockdown PC9 cells and control cells, and found that knockdown of EGFL7 partially blocked its late response, accelerated the decline of Notch NTM regions and suppressed NOTCH signaling as measured by NCID compared with the control (Fig. 6B). Meanwhile, knockdown of EGFL7 downregulated the expression of c-Myc, an effector tightly regulated by NOTCH signaling [47], compared with the control (Fig. 6B). Besides, our data showed that inhibition of NOTCH signaling pathway by dibenzazepine (DBZ) enhanced the antitumor effect of osimertinib in PC9 cells, mimicking the effect of EGFL7 knockdown on EGFR inhibitors (Fig. 6C). Altogether, these data further support our hypothesis.

The above results showed that knockdown of EGFL7 repressed NOTCH signaling and the expression of its downstream effector c-Myc, suggesting the positive regulatory effect of NOTCH signaling on c-Myc. This was also supported by our data showing that combined treatment of osimertinib and NOTCH inhibitor DBZ or DAPT (N-[N-(3, 5-difluorophenacetyl)-l-alanyl]-s-phenylglycinet-butyl ester) decreased the expression of c-Myc compared with osimertinib treatment alone (Fig. 6D). While combined treatment of osimertinib and DBZ or DAPT did not show further inhibitory effect on the phosphorylation of ERK (Fig. 6D). These results provide strong evidence to support the positive regulatory effect of NOTCH cascade on c-Myc. This was not consistent with a previous study indicating that NOTCH signaling suppresses the expression of c-Myc and its downstream targets during the formation of arteries [47]. To address this contradiction, we searched GEO database and extracted all published data of manipulating NOTCH signaling. Next, we performed Gene set enrichment analysis(GSEA) with Myc-related gene sets from MSigDB [29], and demonstrated the two-faced regulatory effect of NOTCH signaling on c-Myc expression (Fig. 6E, F), which was consistent with the above contradictory results. Based on the above results, we conclude that EGFL7 buffers antitumor effect of EGFR inhibitors by activating NOTCH signaling and upregulating c-Myc.

### Blocking EGFL7/NOTCH signaling pathway improves the antitumor efficacy of EGFR inhibitors in irreversible resistant phase

**Fig. 7:**
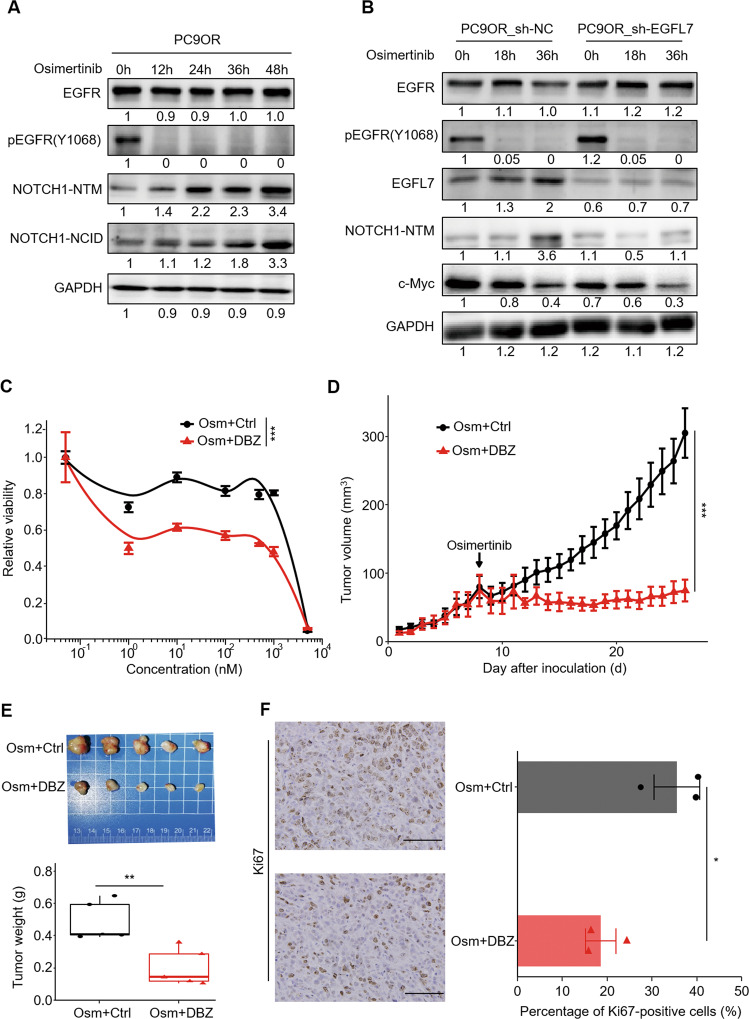
EGFL7/NOTCH signaling pathway promotes resistance to EGFR inhibitors in irreversible resistant phase. **A** Western bolt analysis showing the levels of EGFR, pEGFR, NOTCH1-NTM and NOTCH1-NCID in PC9OR cells treated with 2 µM osimertinib (Osm) for the indicated time points. **B** Western bolt analysis showing the levels of EGFR, pEGFR, EGFL7, NOTCH1-NTM and c-Myc in EGFL7-knockdown PC9OR cells and control cells treated with 2 µM osimertinib (Osm) for the indicated time points. GAPDH was set as a loading control. **C** Survival curves of PC9OR cells treated with a combination of different concentrations of osimertinib (Osm) and vehicle control (DMSO) or NOTCH signaling inhibitor dibenzazepine (DBZ) for 72 h. Data present as average of four identically wells of percentage of surviving cells to vehicle-treated control. **D** Growth curves of tumor xenografts derived from PC9OR cells treated with a combination of 3 mg/kg osimertinib (Osm) and 1 mg/kg DBZ or vehicle control (DMSO). **E** Images of xenograft tumors with the indicated treatments (upper panel) and statistical analysis of tumor weight (lower panel). **F** Left panel showing the representative images of Ki67 staining in xenograft tumors with the indicated treatments, and right panel showing statistical analysis of the percentage of Ki67 positive cells. Scale bar: 100 µm. The data in **E** are average value of five biological replicates. The median line in **E** represents median value. The top and bottom of box in **E** are 25th and 75th percentiles. The data in left panel (**F**) show the representative images of three biological replicates, and the data in right panel (**F**) show the average of three biological replicates. Data are shown as mean ± SEM. *p* values in **C**, **D** are calculated by two way ANNOVA, while *p* values in **E**, **F** are calculated by unpaired two-tailed Student’s *t* test (*p < 0.05; **p < 0.01; ***p < 0.001).

We validated the above mechanism in osimertinib-resistant PC9OR cells. The results showed that, although PC9OR cells lacked the decline phase of Notch1 NTM regions (Fig. 7A), knockdown of EGFL7 similarly blocked the increase of Notch1 NTM and NCID regions compared with the control, thereby accelerating the decline of c-Myc (Fig. 7B). Consistently, the pharmaceutical inhibition of NOTCH signaling by DBZ significantly increased the response of PC9OR cells to EGFR inhibitor osimertinib compared with the control, as measured by 26% decrease in the AUC (Fig. 7C). Similarly, DBZ administration rendered PC9OR cell-derived xenograft tumors more sensitive to osimertinib therapy, compared with the control, as measured by tumor growth rate (Fig. 7D), tumor weight (Fig. 7E) and the levels of proliferative marker Ki67 (Fig. 7F). These data further support that EGFL7/NOTCH signaling drives resistance to EGFR kinase inhibition.

## Discussion

**Fig. 8:**
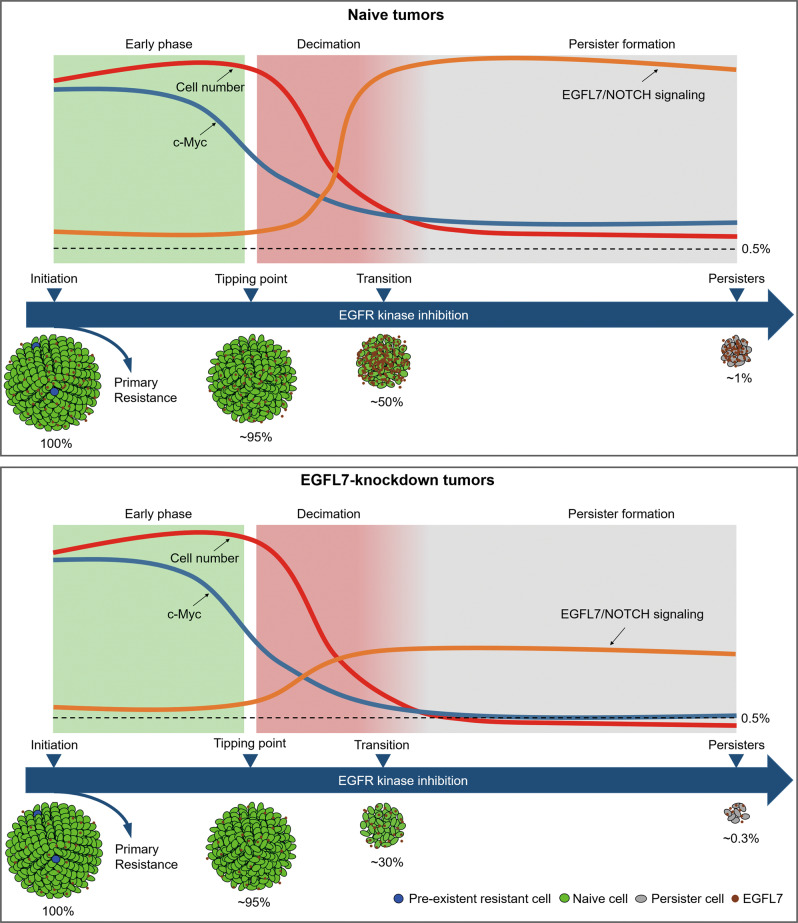
A model for the transition from sensitive cells to persister cells during EGFR kinase inhibition. The cartoon depicts dynamic process as EGFR kinase inhibition elongates. Phenotypically, cells first remain nearly normal then experience rapid number decline and finally a small fraction transform into persister cells. Accordingly, the activity of EGFL7/NOTCH signaling is low in the first stage, increasing during the decimation phase and maintain high since then, decelerating the decline of c-Myc and ultimately contributing to the formation persister cells. Knockdown of EGFL7 largely disturbs this process and results in a lower fraction of persister cells.

Our analysis implicates a cell-autonomous model of entry into a drug-tolerant state which has been regarded as the origins of subsequent resistant states, and this process is driven by EGFL7 (Fig. 8). In this model, we arbitrarily divide the response of sensitive cancer cells to EGFR kinase inhibition into three phases. In early phase, EGFR kinase inhibition only affects a relatively small part of cancer cells and keeps other parts including pathways related with death intact. Thus, cancer cells remain in almost normal state with little decrease in cell vigor until a tipping point comes. In second phase, the influence of EGFR kinase inhibition extends to almost the whole cancer cell system, leading to distorted biological processes and eventually inducing cell death. Phenotypically, the vigor of cancer cell plunges and cancer cells are decimated. However, as any stable system, inhibition of EGFR also elicits some processes buffering its killing effect simultaneously. Here, the responsive increase of EGFL7 decelerates the decrease of c-Myc by activating NOTCH signaling, thereby promoting a higher proportion of cells entering the next phase. During third phase, the activated EGFL7/NOTCH signaling maintains low level of c-Myc expression and gradually counters killing effect of EGFR kinase inhibition, and cancer cells are transitioning to drug-tolerant persister cells. Ultimately, a small but relatively consistent proportion of sensitive cells transform into persister cells, while knockdown of EGFL7 disturbs the process, thereby causing a significant decrease in proportion of surviving cells.

EGFL7 has the traits of early onset and rendering a relative small selective advantage during tumor’s evolution to drug resistance, which is similar with those driver genes identified in tumorigenesis have [48]. Thus, we define EGFL7 as a driver gene for resistance to EGFR inhibitors. On the contrary to tumorigenesis, the evolution to resistance identified here somehow mimics cell differentiation during which cells tune their function from proliferation to facilitate differentiation [49], supporting that drug-tolerant state is a transform of naive state rather than a selection of previous existent state. Similarly, the treatment of MAPK inhibitors has also been linked with differentiation [50]. This will complement the model of acquired resistance and may benefit targeted therapy.

The early phase of acquired resistance fits well with perturbation experiments, which is a dynamic system with several distinct traits. First, responses to perturbation are divided into two opposite categories. One leads to different forms of cell death, while the other counters the effect of perturbation. Of note, these two categories happen simultaneously. Thus, in sensitive situation where most population is going to death, the top responsive genes, often defined as differentiated genes, may well fall in the first category. Second, most mechanisms are defined within thermodynamics. That is, we seldom consider the time cost by signal transduction, which may fit well with stable systems. However, the mechanism underlying in a dynamic system must take time cost as a prior. Finally, regulatory networks behind cell death are still not well understood. Although we have clarified signaling pathways behind main forms of cell death, the exception always exists [51]. Thus, the genes reflecting persister status should be confirmed in specific situations. Considering the above, we screened differentially expressed genes between naive and stable resistant status, confirmed with published data on EGFR kinase inhibition and took the use of turnover rates as criteria to identify underling molecular processes and selected c-Myc as a direct reflection of phenotype [52].

Our data show that the formation of persister cells to EGFR inhibitors is at least partly driven by the adaptive increase of EGFL7, and demonstrate that the adaptive upregulation of EGFL7 is regulated by the depressed NMD pathway. In naive status, the transcripts are rapidly decayed via NMD pathway, while stress somehow dampens NMD pathway, thus those transcripts entrance into translation system to help cell survive corresponding stress. Moreover, previous studies have demonstrated that alternative TISs are conserved in vertebrates [53, 54], and cells prefer alternative TISs under stress [41, 55, 56]. In addition, compared with depression mechanism of KEAP-NRF2 system, NMD-RNA system will consume much less energy [57]. Phenotypically, NMD has also been linked with stress response and differentiation [39]. These observations, taken together, imply the physiological importance of this mechanism in managing stress.

Our results implicate the existence of EGFL7/NOTCH/c-Myc signaling pathways. As supported, the direct interaction between EGFL7 and NOTCH1 has been validated by antibody array [58], in addition to interaction database. The EMI and EGF like domain, located in the C terminus of EGFL7, mediates this interaction [45]. Although their interaction leads to contradictory results [45, 59], this can be explained by the complexity of NOTCH signaling [46]. Another evidence is that the RGD domain of EGFL7 has been demonstrated to be a modulator of NOTCH signaling [60]. Additionally, the RGD domain of EGFL7 is important to angiogenesis [61] and EGFL7 has been linked to NOTCH and integrin signaling pathways besides cancers [62]. Besides, the relationship between NOTCH signaling and c-Myc seems more complex, further supporting the two-faced role of NOTCH signaling in different context. On one hand, NOTCH signaling is highly context-dependent [46]. Consistently, even HES1, a canonical downstream gene of NOTCH signaling, fails to be filtered out in Notch stimulation experiments by canonical Notch ligands [63]. On the other hand, several known regulatory factors fail to explain the suppression of c-Myc in treatment-persister cells [52]. These observations implicate that there exists at least two opposite pathways linking NOTCH signaling with the expression of c-Myc. Intriguingly, a recent study about synthetic biology has demonstrated that such paradoxical design can provide robust control [64]. These seemingly contradict results showed here, taken together, may be common design used by cells for intricate regulation.

In conclusion, the present study complements current model of acquired drug resistance which states cells first enter a reversible quiescent state, and then develop different mechanisms that help them thrive in the presence of drugs. Although current model echoes clinical observations of “drug holiday” and diverse resistant mechanism presented during clinical therapy [15], it falls short in guiding treatment without further details. For example, inhibition of c-Myc activity fails to increase the proportion of chemotherapy-induced persister cells which show decreased expression of c-Myc [52]. In contrast, depressing NOTCH signaling indicated by our analysis has been proved as an appealing method to tackle drug resistance in different in vitro models [65–67]. However, key nodes identified here fail to explain the metabolism change in early phase of resistance [31], suggesting that a complex picture need further studies.

## Supplementary information

Supplementary Materials

Original Data File

reproducibility checklist

## Data Availability

All processed bulk RNA-seq data have been deposited with the Gene Expression Omnibus under accession code GSE201549. Previously published transcriptomic data reanalyzed here are available with accession code listed in methods.

## Code availability

All computer code used are slightly modified from specific user’s guides and these guides can be accessed from corresponding R packages.
