# Supplementary material for: EGFL7 drives the evolution of resistance to EGFR inhibitors in lung cancer by activating NOTCH signaling
Source: Cell Death Dis. 2022 Oct 29;13(10):910. doi: 10.1038/s41419-022-05354-y (PMC9617940; doi:10.1038/s41419-022-05354-y)
Supplement: Supplementary file 1 — Supplementary Materials [file 41419_2022_5354_MOESM1_ESM.docx]

**Supplementary Materials**

Supplementary Table S1. Sequence information of nucleotides used in this study

| Items | Sequences (5’-3’) |
| --- | --- |
| si-EGFL7-1 | GTGGATGAATGCAGTGCTA |
| si-EGFL7-2 | GCGAGCAGATTTCCTTCCT |
| sh-EGFL7 | GTGGATGAATGCAGTGCTA |

Supplementary Table S2. Gene sets used in GSEA^1^

| Gene set | Systematic Name | Source publication (PMID) |
| --- | --- | --- |
| COLLER_MYC_TARGETS_DN | M19620 | 10737792 |
| COLLER_MYC_TARGETS_UP | M5955 | 10737792 |
| DANG_MYC_TARGETS_DN | M17557 | 14519204 |
| DANG_MYC_TARGETS_UP | M6506 | 14519204 |
| DANG_REGULATED_BY_MYC_DN | M2310 | 14519204 |
| DANG_REGULATED_BY_MYC_UP | M18501 | 14519204 |
| ELLWOOD_MYC_TARGETS_DN | M6951 | 14522256 |
| ELLWOOD_MYC_TARGETS_UP | M2607 | 14522256 |
| LEE_LIVER_CANCER_MYC_DN | M18496 | 15565109 |
| LEE_LIVER_CANCER_MYC_UP | M2742 | 15565109 |
| SCHUHMACHER_MYC_TARGETS_DN | M969 | 11139609 |
| SCHUHMACHER_MYC_TARGETS_UP | M12243 | 11139609 |
| YU_MYC_TARGETS_DN | M3923 | 16382050 |
| YU_MYC_TARGETS_UP | M1249 | 16382050 |

^1^GSEA, Gene Set Enrichment Analysis

Supplementary Table S3. Antibodies used in this study

| Antibodies | Source | Identifiers |
| --- | --- | --- |
| Notch1 (C44H11) Rabbit mAb | Cell Signaling Technology | CAT#3268 |
| Cleaved Notch1 (Val1744) (D3B8) Rabbit mAb | Cell Signaling Technology | CAT#4147 |
| p44/42 MAPK (Erk1/2) (137F5) Rabbit | Cell Signaling Technology | CAT#4695 |
| Phospho-p44/42 MAPK (Erk1/2) (Thr202/Tyr204) (D13.14.4E) XP® Rabbit mAb | Cell Signaling Technology | CAT#4370 |
| EGF Receptor (D38B1) XP® Rabbit mAb | Cell Signaling Technology | CAT#4267 |
| Phospho-EGF Receptor (Tyr1068) (D7A5) XP® Rabbit mAb | Cell Signaling Technology | CAT#3777 |
| Recombinant Anti-c-Myc antibody [Y69] | Abcam | CAT#ab32072 |
| Recombinant Anti-EGFL7 antibody [EPR22603-113] | Abcam | CAT#ab256451 |
| Rabbit polyclonal Anti-GAPDH antibody | Abcam | CAT#ab8245 |
| Mouse Monoclonal Anti-RENT1 antibody | Santa Cruz Biotechnology | CAT#sc-393594 |
| Mouse Monoclonal Anti-RENT2 antibody | Santa Cruz Biotechnology | CAT#sc-374230 |
| Mouse Monoclonal Anti-RENT3 antibody | Santa Cruz Biotechnology | CAT#sc-398821; |

Supplementary Table S4. Patient information from TCGA database

| \| bcr_patient_barcode \| EGFR^1^ inhibitors received \| treatment_best_response \| Transcriptome file ID \| \| --- \| --- \| --- \| --- \| \| TCGA-05-4402 \| Erlotinib \| Complete Response \| 77c6e9b5-926e-4eec-be86-efc6627bda2f \| \| TCGA-05-5425 \| Gefitinib \| Clinical Progressive Disease \| 920d5952-c102-47c4-9675-6340de8afae5 \| \| TCGA-50-5055 \| Tarceva \| Clinical Progressive Disease \| 7353ad52-e10c-4712-a2b7-43d01ca1821c \| \| TCGA-53-7624 \| Erlotinib \| Clinical Progressive Disease \| 193ddc3f-b7fc-4f3a-a4e8-7e7e74734b7d \| \| TCGA-55-6982 \| Tarceva \| Clinical Progressive Disease \| ecd9091a-4d46-4a74-ada2-24c4b2e64526 \| \| TCGA-55-8513 \| Tarceva \| Clinical Progressive Disease \| 82b7b19d-162c-417e-b38b-d1ac77a4969d \| \| TCGA-64-5778 \| Tarceva \| Clinical Progressive Disease \| 2dcc20e1-8634-448b-823a-deb3fc038651 \| \| TCGA-73-4675 \| Tarceva \| Clinical Progressive Disease \| 11fa2ef9-728c-4e7d-a458-f781648c4064 \| |
| --- | --- | --- | --- | --- | --- | --- | --- | --- | --- | --- | --- | --- | --- | --- | --- | --- | --- | --- | --- | --- | --- | --- | --- | --- | --- | --- | --- | --- | --- | --- | --- | --- | --- | --- | --- | --- |

EGFR, epidermal growth factor receptor

Supplementary Table S5*.* Interactome of EGFL7^1^ in HuRI^2^ database

| Interactor A (gene symbol) | Interactor B (gene symol) |
| --- | --- |
| EGFL7 | MID2 |
| EGFL7 | HSD3B7 |
| EGFL7 | HOXA1 |
| EGFL7 | SPAG8 |
| EGFL7 | GNE |
| EGFL7 | HEXIM2 |
| EGFL7 | NOTCH1 |
| EGFL7 | HGS |
| EGFL7 | RASSF10 |

^1^EGFL7, EGF like domain multiple 7; ^2^ HuRI, The Human Reference Interactome

Supplementary Table S6. Interactome of EGFL7^1^ in BioGRID^2^ database

| Interactor A (gene symbol) | Interactor B (gene symbol) | Interactor A (gene symbol) | Interactor B (gene symbol) |
| --- | --- | --- | --- |
| EGFL7 | A2M | EGFL7 | LRP3 |
| EGFL7 | ADAM21 | EGFL7 | LTBR |
| EGFL7 | ADAM33 | EGFL7 | LY86 |
| EGFL7 | ASGR2 | EGFL7 | MFAP5 |
| EGFL7 | C7orf34 | EGFL7 | MID2 |
| EGFL7 | CCL3 | EGFL7 | NELL1 |
| EGFL7 | Cenpe | EGFL7 | NELL2 |
| EGFL7 | CLTC | EGFL7 | NOTCH1 |
| EGFL7 | CRLF1 | EGFL7 | NOTCH2 |
| EGFL7 | DGCR2 | EGFL7 | NOTCH3 |
| EGFL7 | DLK1 | EGFL7 | NOTCH4 |
| EGFL7 | Eef1a1 | EGFL7 | NRSN1 |
| EGFL7 | EFEMP1 | EGFL7 | NUFIP2 |
| EGFL7 | EGFL8 | EGFL7 | OLFM2 |
| EGFL7 | ENG | EGFL7 | ORF8 |
| EGFL7 | FBLN5 | EGFL7 | PIGT |
| EGFL7 | FN1 | EGFL7 | PLA2G10 |
| EGFL7 | FXR1 | EGFL7 | PLAUR |
| EGFL7 | FXR2 | EGFL7 | PNMA2 |
| EGFL7 | GFI1B | EGFL7 | PRG2 |
| EGFL7 | GNE | EGFL7 | PROZ |
| EGFL7 | GRN | EGFL7 | RAB6B |
| EGFL7 | HEXIM2 | EGFL7 | RASSF10 |
| EGFL7 | HGS | EGFL7 | RCN1 |
| EGFL7 | HLA-DPA1 | EGFL7 | ROCK2 |
| EGFL7 | HOXA1 | EGFL7 | SLURP1 |
| EGFL7 | HPN | EGFL7 | SMARCD1 |
| EGFL7 | HSD3B7 | EGFL7 | Smn1 |
| EGFL7 | HSPA5 | EGFL7 | SPAG8 |
| EGFL7 | IFI30 | EGFL7 | SPINT2 |
| EGFL7 | KCMF1 | EGFL7 | ST14 |
| EGFL7 | KIF14 | EGFL7 | TRIM25 |
| EGFL7 | KIF20A | EGFL7 | UBR3 |
| EGFL7 | KLRG2 | EGFL7 | UBR4 |
| EGFL7 | LDLRAD1 | EGFL7 | VHL |
| EGFL7 | LIPH | EGFL7 | VWCE |
| EGFL7 | LMBR1L | EGFL7 | WDR76 |

^1^EGFL7, EGF like domain multiple 7; ^2^ BioGRID, The Biological General Repository for Interaction Datasets


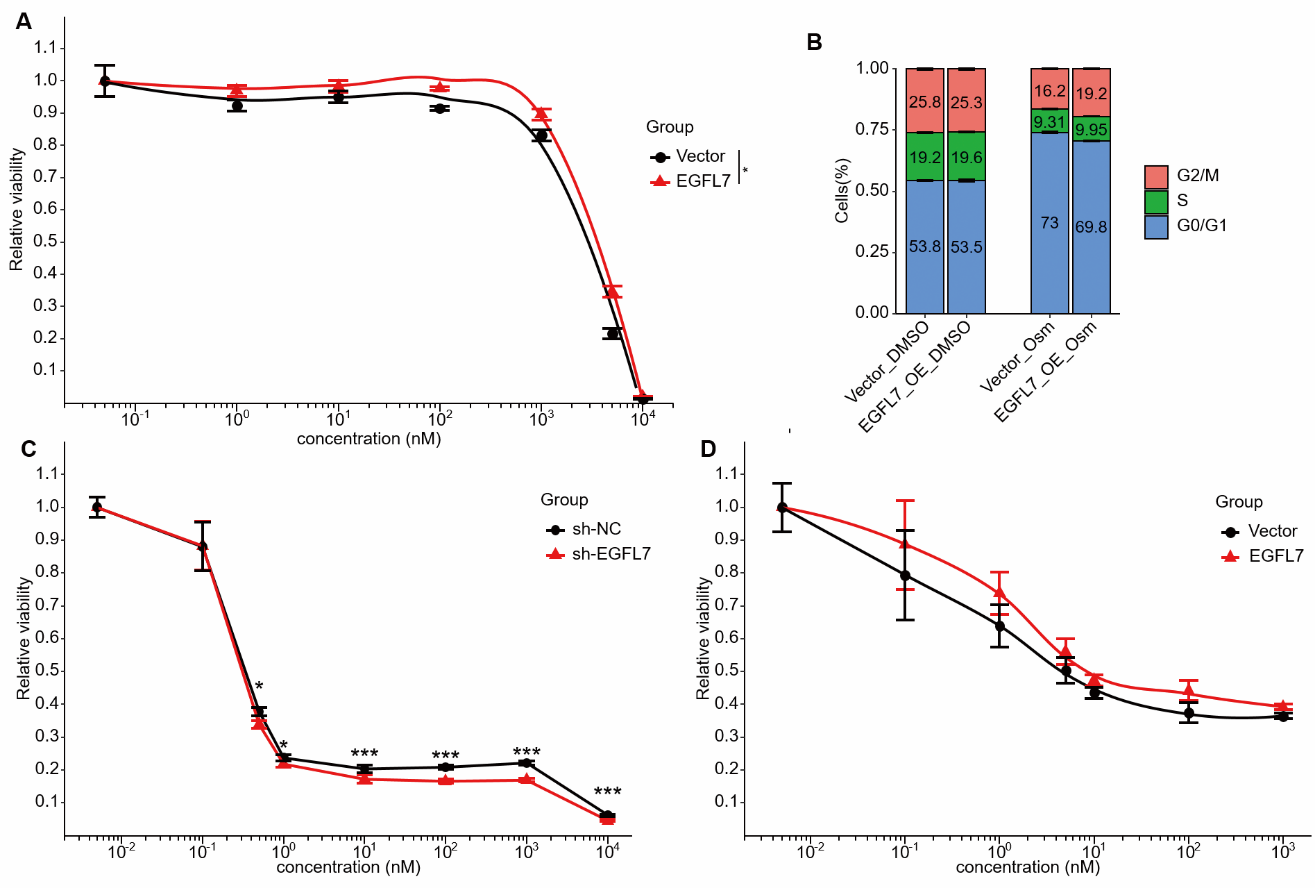


Supplementary Fig. S1 EGFL7 induces resistance to EGFR inhibitors in sensitive phase. A Survival curve of osimertinib-resistant PC9 (PC9OR) cells with the indicated expression plasmids in different concentration of osimertinib treatment for 72 h. Data present as average of 4 identically wells of percentage of surviving cells to 6 wells of vehicle-treated control. B Cell cycle distribution of PC9OR cells with the indicated expression plasmids treated with 3 µM osimertinib or vehicle. Data present as mean ± standard deviation (SD) of 3 biological replicates. Survival curves of PC9 cells with the indicated sh-RNAs C or expression plasmids D in different concentrations of osimertinib treatment for 72 h. Data present as average of 4 identically wells of percentage of surviving cells to vehicle-treated control. Data are shown as mean ± SEM. *P* values are calculated by two way ANNOVA (*, *P* <0.05; ***, *P* <0.001).


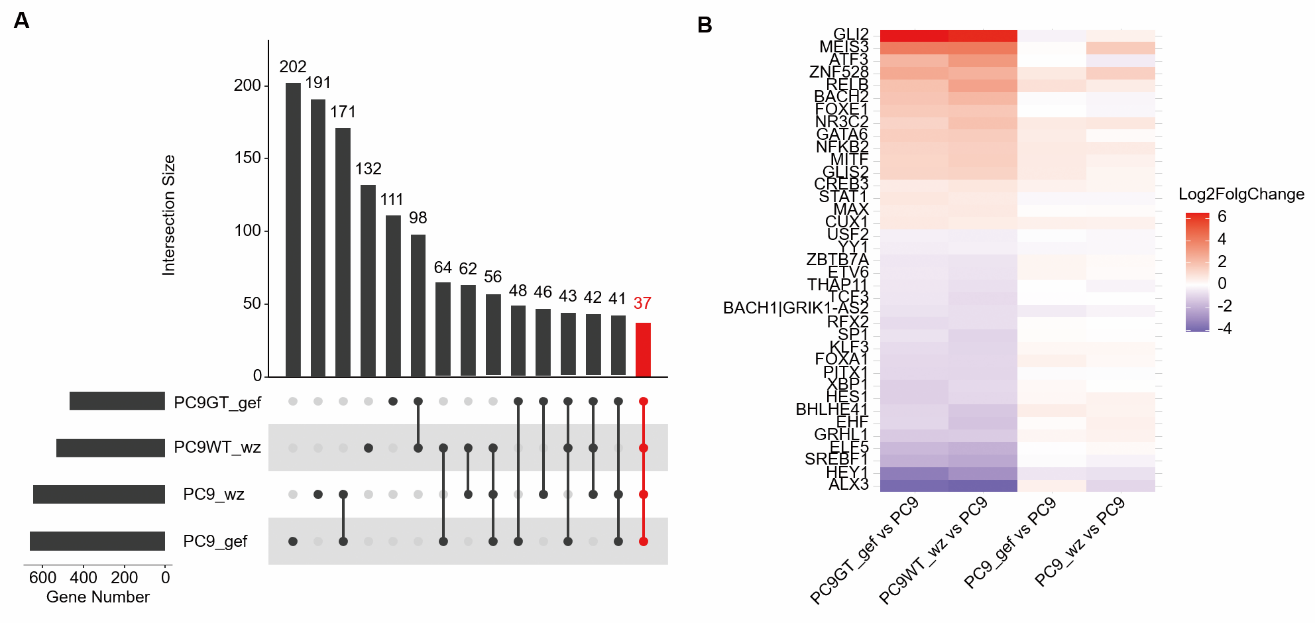


Supplementary Fig. S2 Upregulated genes in late responsive period. A Upset plot representing the changed genes in the indicated EGFR inhibitors-treated PC9 tolerant cells or unchanged genes in the indicated EGFR inhibitors-treated PC9 parental cells compared with PC9 parental cells. Red shapes presenting desired gene set. B Relative expression of genes from panel A.
