# Supplementary figures and images for: EGFL7 drives the evolution of resistance to EGFR inhibitors in lung cancer by activating NOTCH signaling
Source: Cell Death Dis. 2022 Oct 29;13(10):910. doi: 10.1038/s41419-022-05354-y (PMC9617940; doi:10.1038/s41419-022-05354-y)

Figure 4E

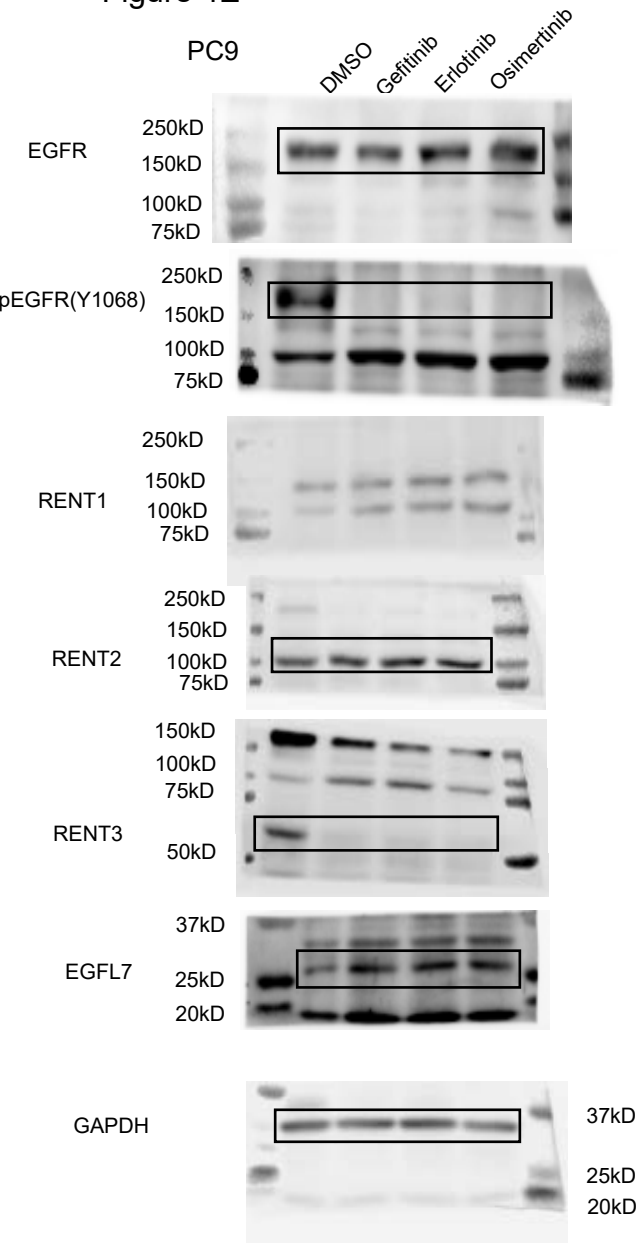

Figure 4F

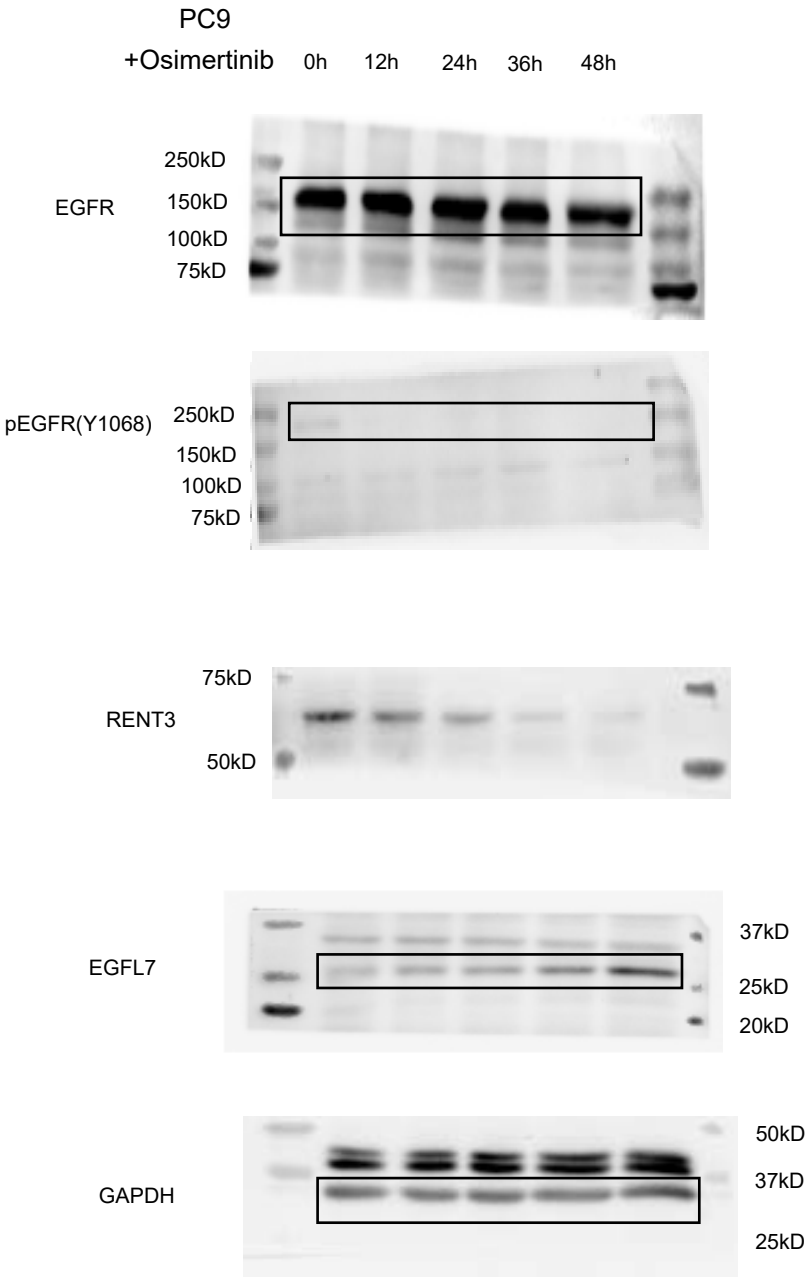

Figure 6A

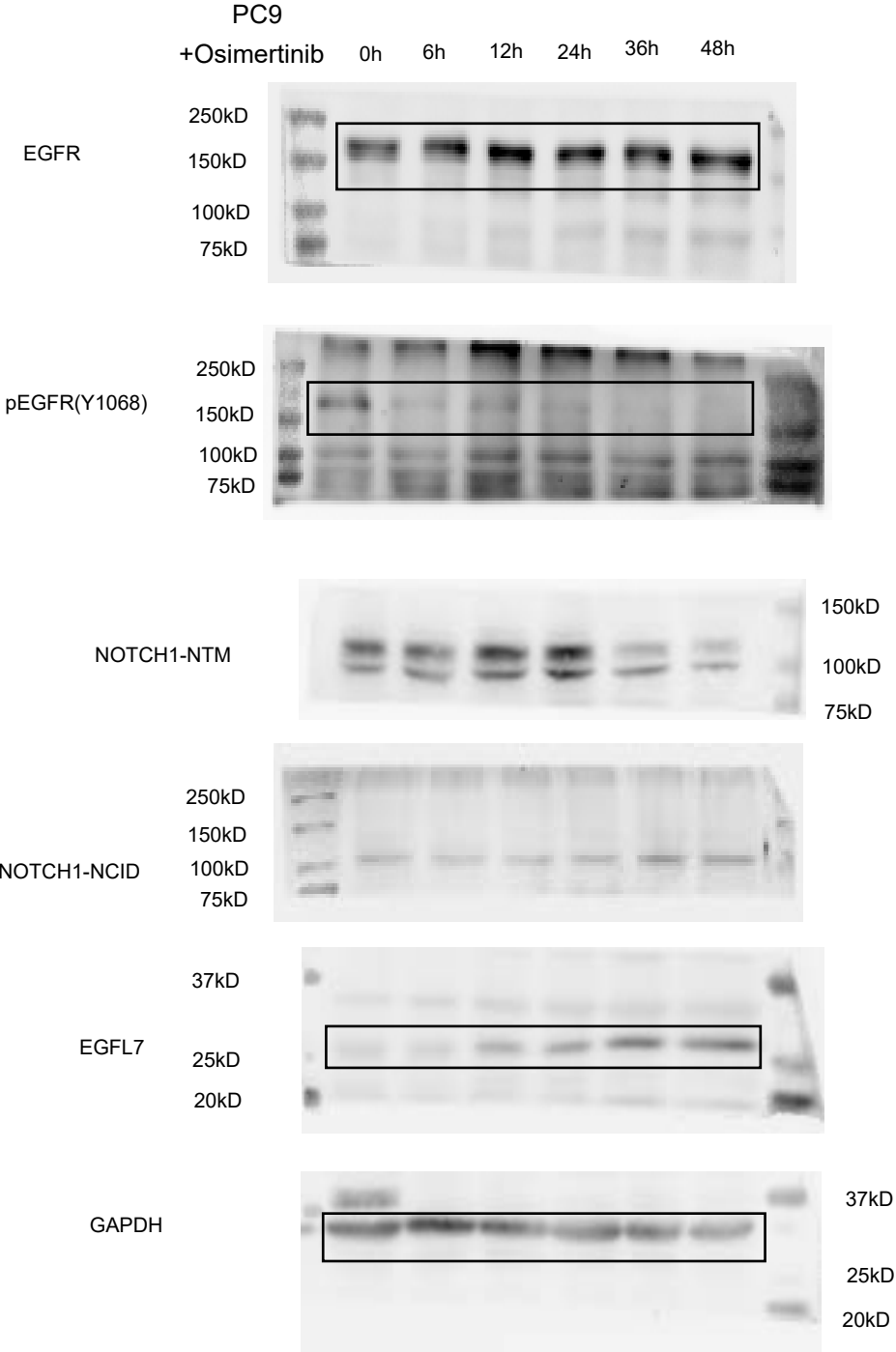

Figure 6B

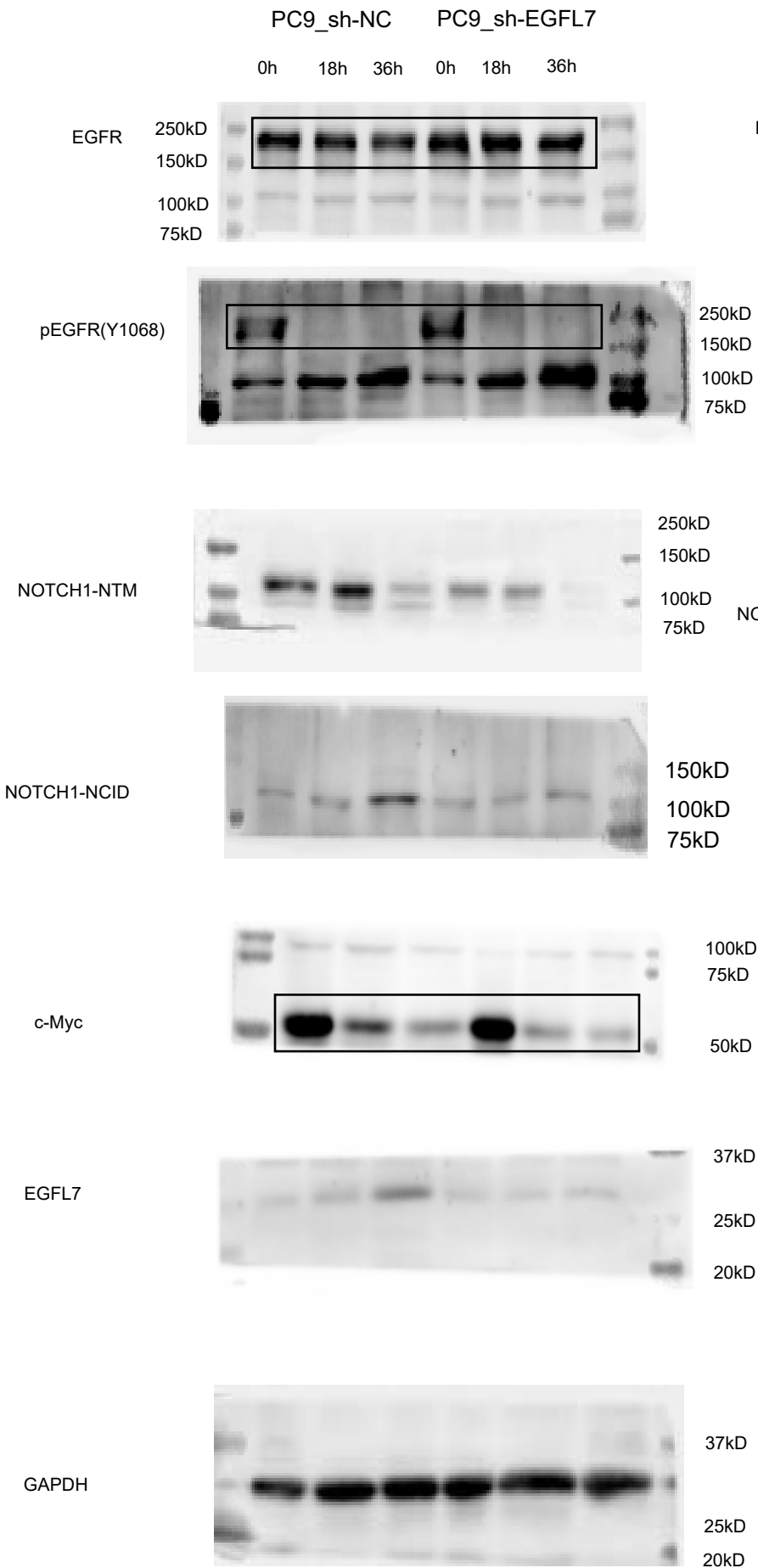

Figure 6D

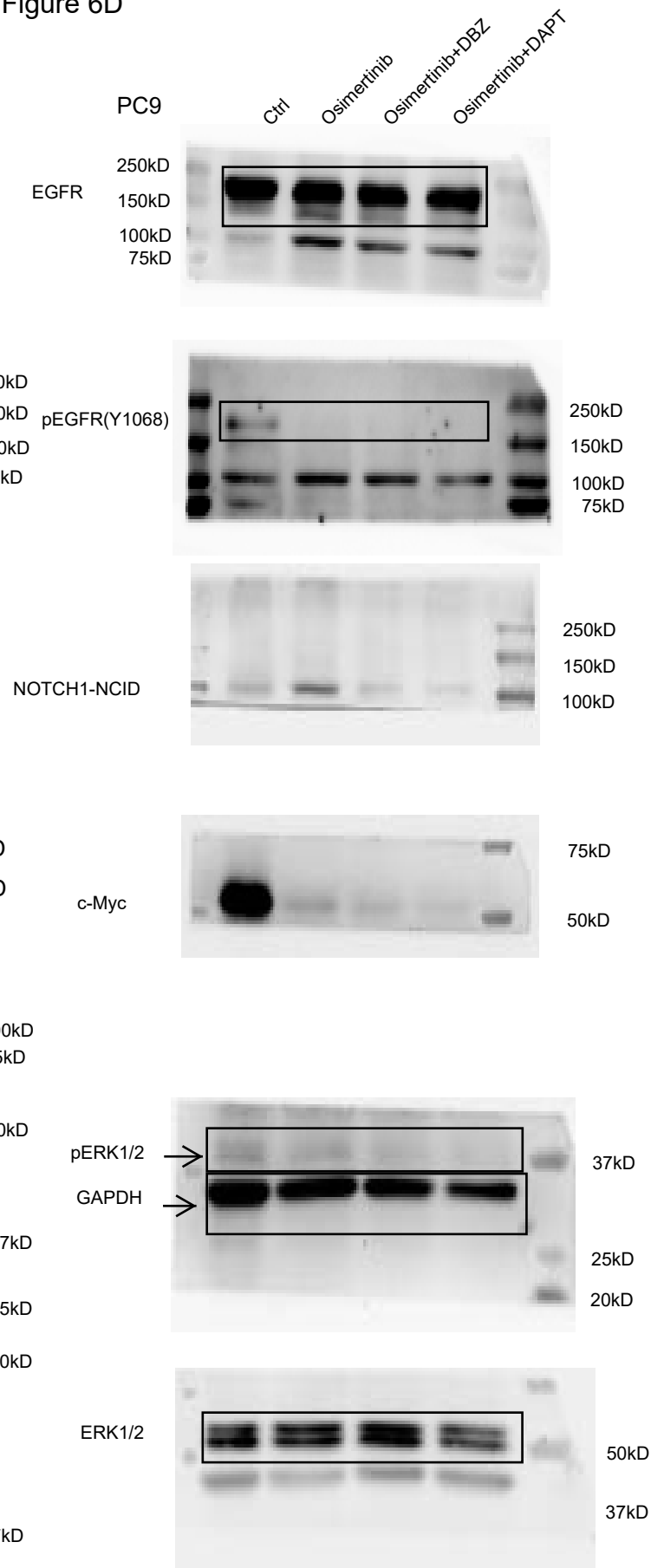

Figure 7A

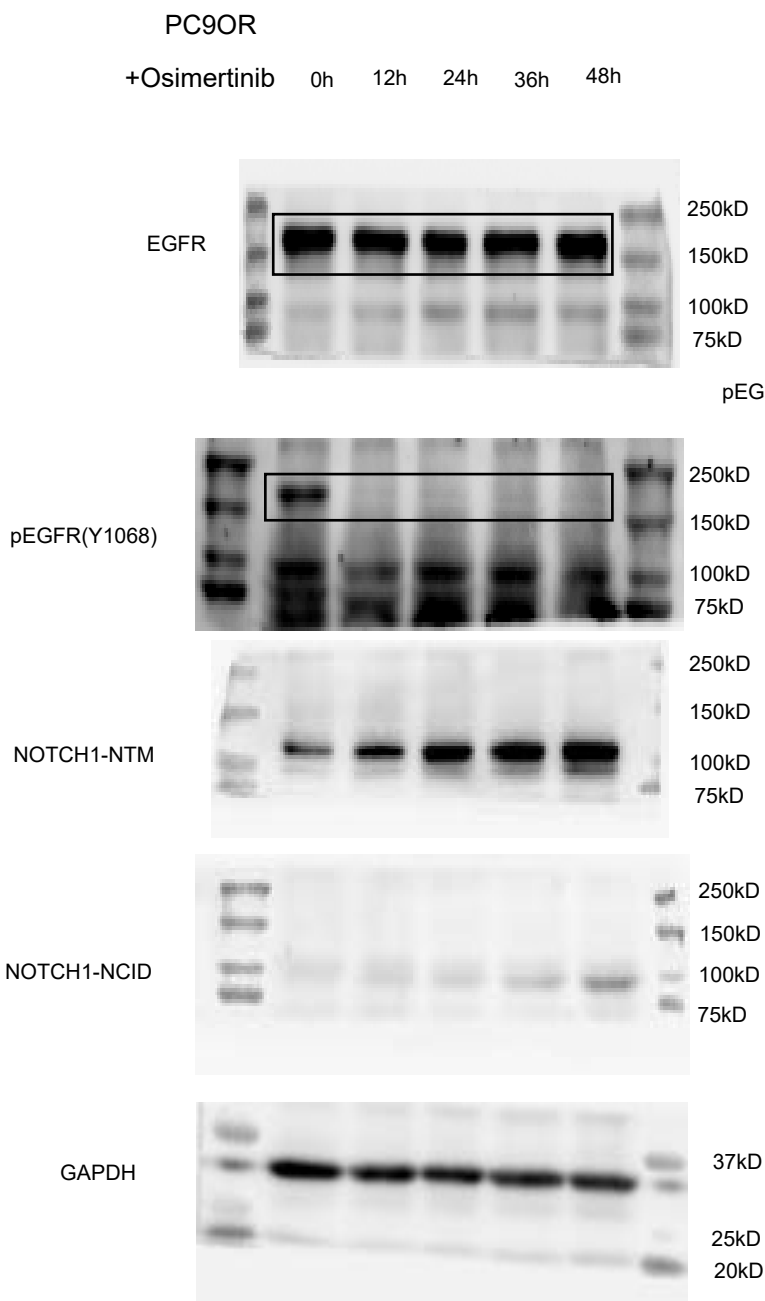

Figure 7B

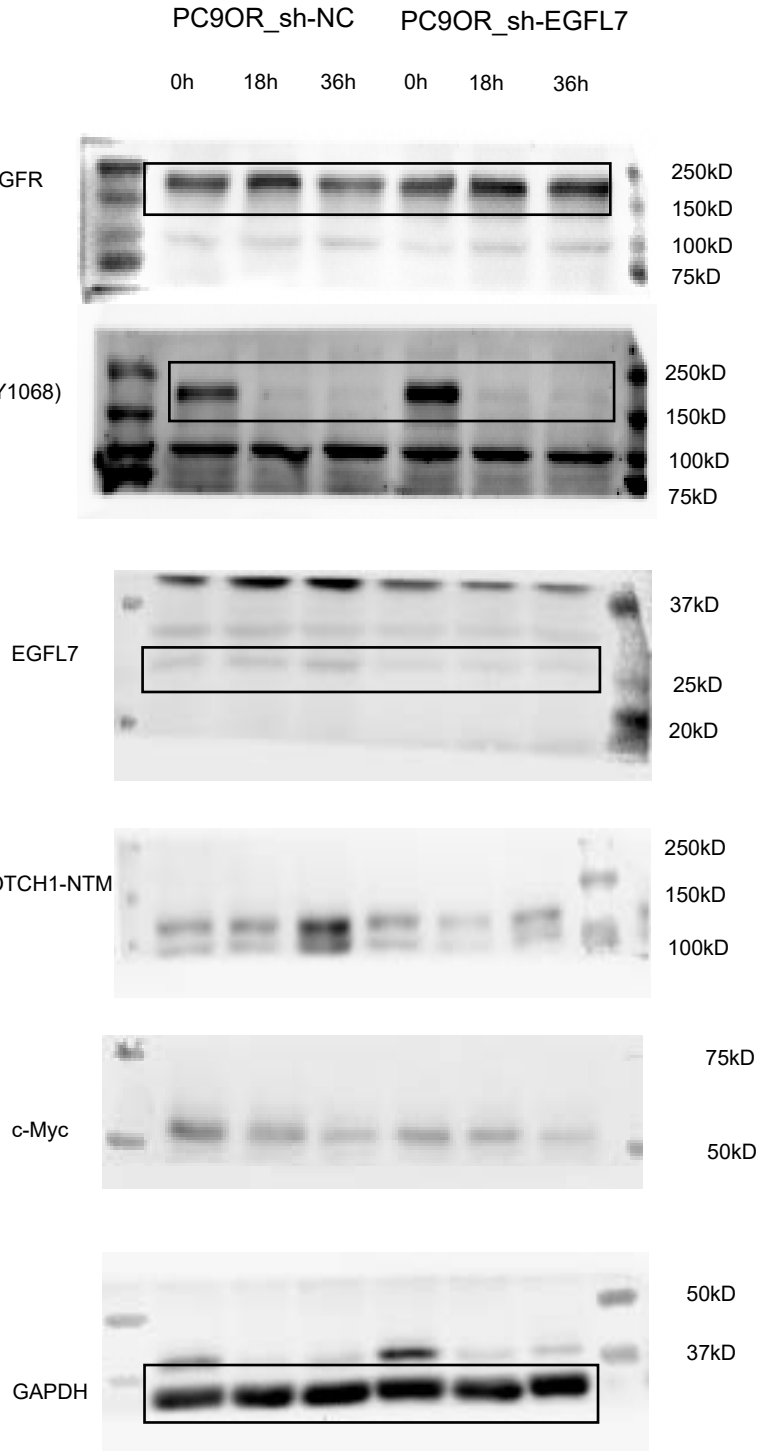

Supplement: Supplementary file 2 — Original Data File [file 41419_2022_5354_MOESM2_ESM.pdf]
